# Peripheral Regulatory T Cells Take the Road Less Traveled

**DOI:** 10.1371/journal.pbio.1000065

**Published:** 2009-03-03

**Authors:** Kira Heller

T cells serve as key weapons in the mammalian immune system's arsenal, defending the host against invading pathogens such as bacteria and viruses. But sometimes these powerful weapons attack host tissues, wreaking havoc in the form of autoimmune diseases like lupus erythematosus and multiple sclerosis. The immune system uses two main mechanisms to prevent such attacks: negative selection, in which T cells with a high affinity for host peptides are sentenced to death before they leave the thymus (where T cells develop), and dominant tolerance, in which a specialized subpopulation of T cells modulate the activity of T cells and other lymphocytes against host tissue and pathogens. These regulatory T (T_reg_) cells, which help prevent autoimmune disease by dampening immune responses, are characterized by expression of the Foxp3 transcription factor and are mostly derived from the thymus. However, recent research indicates that a small percentage of CD4^+^Foxp3^+^ T_reg_ cells may develop not in the thymus, but in the peripheral lymphoid organs (lymph nodes and spleen). Although it's possible that T_reg_ cells produced in either the thymus or the periphery perform different tasks, the two populations appear indistinguishable in wild-type mice.

To find out what might set peripheral T_reg_ cells apart from their thymic counterparts, Michael Barnes and colleagues induced genetic mutations in mice using the chemical N-ethyl-N-nitrosourea (ENU). By screening for mice with defects in T cell development and function, the researchers identified a mutation in a gene called Carma1 (which was previously shown to play a key role in T and B cell signaling) that caused mice to have essentially no thymic T_reg_ cells. However, they did have a small population of T_reg_ cells in their spleen, lymph nodes, and colon, indicating that T_reg_ cells might still be developing in the peripheral lymphoid organs. In vitro, these peripheral T_reg_ cells could be induced to express Foxp3 and proliferate in response to the cytokines TGFβ and IL-2, which are produced at increased levels during infection.

**Figure:**
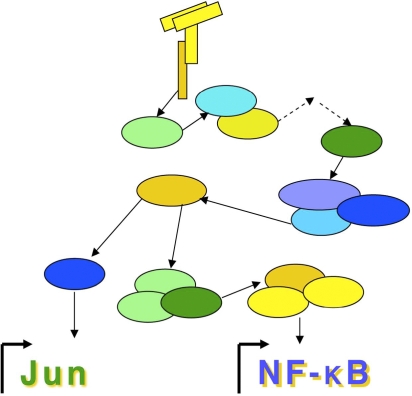
CARMA1 (purple oval), part of a signaling pathway required for regulatory T cell development in the thymus, links T cell receptor ligation to activation of the transcription factors NF-κB and Jun.

Unlike most mice with mutations that impair T_reg_ cell development, the Carma1-mutant mice did not exhibit any obvious signs of autoimmune disease, such as an enlarged spleen or chronic inflammation. This indicates that although T_reg_ cells were not being produced in the thymus, dominant tolerance was not entirely absent because of the presence of the peripheral T_reg_ cells. To see if the T_reg_ cells observed in the periphery of *Carma1*-mutant mice were capable of expanding in response to pathogens, the researchers infected the mice with mouse cytomegalovirus (MCMV). Although Foxp3 expression and massive T_reg_ expansion were observed in the periphery 14 days after infection, no Foxp3^+^ cells were detected in the thymus, indicating that the expanded T_reg_ cells arose from the small pool of peripheral T_reg_ cells.

Why might there be distinct thymic/Carma1-dependent and peripheral/Carma1-independent pathways for inducing Foxp3 expression and thus committing CD4^+^ cells to the T_reg_ cell lineage? By having a pool of T_reg_ cells with two potential fates, the immune system gains flexibility in how it induces and modulates its responses. Thus, while under normal conditions, thymic T_reg_ cells might be automatically generated and proliferate to compose the majority of the T_reg_ pool, the generation and massive expansion of peripheral T_reg_ cells could be quickly induced by cytokines produced in response to invasion by pathogens. During an immune response, the Carma1-independent pathway could allow some T cells to become T_reg_ cells and expand rapidly, which could protect the host from cross-reactive antipathogen T cells that might otherwise attack self tissue and cause autoimmune disease. For example, impaired immune regulation often leads to the autoimmune disease colitis. That elevated numbers of T_reg_ cells normally reside in the colon and can expand even in the absence of Carma1 suggests that the Carma1-independent pathway may be important for regulating responses to the billions of bacteria that reside in the intestines of healthy mice. On the flipside, however, suppressing the immune response could lead to chronic infection, particularly if T_reg_ cells are localized to pathogen reservoir sites. Indeed, previous research has shown that some pathogens can manipulate the host immune response to their advantage by promoting the expansion of the T_reg_ cells that prevent their eradication by the immune system. By learning more about how T_reg_ cells are generated and their role in the immune response, researchers might one day be able to manipulate them therapeutically by suppressing their function to fight infection or by enhancing it to prevent autoimmune disease.

